# The Inflammasome Pyrin Contributes to Pertussis Toxin-Induced IL-1β Synthesis, Neutrophil Intravascular Crawling and Autoimmune Encephalomyelitis

**DOI:** 10.1371/journal.ppat.1004150

**Published:** 2014-05-29

**Authors:** Aline Dumas, Nathalie Amiable, Juan Pablo de Rivero Vaccari, Jae Jin Chae, Robert W. Keane, Steve Lacroix, Luc Vallières

**Affiliations:** 1 Axis of Neuroscience, University Hospital Center of Quebec, Quebec, Quebec, Canada; 2 Department of Neurological Surgery, The Miami Project to Cure Paralysis, University of Miami, Miami, Florida, United States of America; 3 Medical Genetics Branch, National Human Genome Research Institute, Bethesda, Maryland, United States of America; 4 Department of Physiology and Biophysics, University of Miami, Miami, Florida, United States of America; 5 Department of Molecular Medicine, Laval University, Quebec, Quebec, Canada; University of Toronto, Canada

## Abstract

Microbial agents can aggravate inflammatory diseases, such as multiple sclerosis (MS) and its animal model, experimental autoimmune encephalomyelitis (EAE). An example is pertussis toxin (PTX), a bacterial virulence factor commonly used as an adjuvant to promote EAE, but whose mechanism of action is unclear. We have reported that PTX triggers an IL-6-mediated signaling cascade that increases the number of leukocytes that patrol the vasculature by crawling on its luminal surface. In the present study, we examined this response in mice lacking either TLR4 or inflammasome components and using enzymatically active and inactive forms of PTX. Our results indicate that PTX, through its ADP-ribosyltransferase activity, induces two series of events upstream of IL-6: 1) the activation of TLR4 signaling in myeloid cells, leading to pro-IL-1β synthesis; and 2) the formation of a pyrin-dependent inflammasome that cleaves pro-IL-1β into its active form. In turn, IL-1β stimulates nearby stromal cells to secrete IL-6, which is known to induce vascular changes required for leukocyte adhesion. Without pyrin, PTX does not induce neutrophil adhesion to cerebral capillaries and is less effective at inducing EAE in transgenic mice with encephalitogenic T lymphocytes. This study identifies the first microbial molecule that activates pyrin, a mechanism by which infections may influence MS and a potential therapeutic target for immune disorders.

## Author Summary

Microbial agents can aggravate inflammatory diseases, such as multiple sclerosis (MS) and its animal model, experimental autoimmune encephalomyelitis (EAE). An example is pertussis toxin (PTX), which is used to promote EAE by an obscure mechanism. We have reported that PTX triggers an IL-6-mediated signaling cascade that increases the number of leukocytes that patrol the vasculature by crawling on its luminal surface. We show here that PTX, through its ADP-ribosyltransferase activity, induces: 1) TLR4 signaling in myeloid cells, leading to pro-IL-1β synthesis; and 2) a pyrin-dependent inflammasome that cleaves pro-IL-1β into its active form. Then, IL-1β stimulates nearby stromal cells to secrete IL-6. Without pyrin, PTX does not induce neutrophil adhesion to cerebral capillaries and is less effective at inducing EAE in mice with encephalitogenic T lymphocytes. This study identifies the first microbial molecule that activates pyrin, a mechanism by which infections may influence MS and a potential therapeutic target for immune disorders.

## Introduction

MS is a T lymphocyte-mediated autoimmune demyelinating disease of the central nervous system (CNS), whose development is dictated by complex interactions between genetic and environmental factors [1], [2]. Among the latter are microbes and their toxins. Although the search for a causative microbial agent has proved fruitless so far, many epidemiological studies have shown associations between infections and MS. For example, the risk of developing MS is at least doubled in individuals with a clinical history of infectious mononucleosis [3]–[11], while the risk of MS exacerbation is higher after common infections of the upper respiratory tract [3], [12]–[17].

Several non-exclusive mechanisms have been proposed to explain the influence of microbial agents on MS [18], [19]. One hypothesis, called bystander activation, states that such agents trigger adjuvant effects that can non-specifically promote the activation of autoreactive lymphocytes and their trafficking across the blood-brain barrier. This idea is supported by the observation that transgenic mice with a large number of myelin-reactive T cells do not develop spontaneous encephalomyelitis when housed in pathogen-free conditions, but do so after injection of bacterial toxins or when raised in a non-sterile environment [20]. Furthermore, recent studies show that the intestinal microbial flora contributes to the development of EAE [21]–[23], the most widely used animal model of MS.

To induce EAE, mice immunized with myelin antigens or transplanted with myelin-reactive T cells are commonly injected with PTX, a hexameric protein produced by the bacteria causing whooping cough and used as a surrogate for infection to increase EAE incidence and severity [24], [25]. The mechanism by which PTX promotes EAE is still not fully understood and seems paradoxical considering the ability of PTX to block leukocyte migration by interfering with chemokine receptor signaling [26]. This effect results from its largest subunit, called S1 or A protomer, which is endocytosed and transported to the endoplasmic reticulum [27], where it catalyzes the ADP-ribosylation of G-protein α_i_ subunits [28], [29], thereby preventing their interaction with G protein-coupled receptors. Another mechanism has been proposed, in which the other subunits that form the B oligomer (i.e., the subunit S5 resting on two dimers, S4–S2 and S4–S3) would interact with transmembrane receptors, leading to the induction of inflammatory signaling pathways [30]. Although this hypothesis remains to be proved, it is indirectly supported by several observations. For example, Kubes and colleagues have reported that PTX stimulates P-selectin expression and leukocyte adhesion on the cerebral endothelium by acting through Toll-like receptor 4 (TLR4) [31]. Furthermore, we have shown that PTX increases the plasma level of IL-6, which in turn increases the expression of endothelial adhesion molecules and chemokines, leading to the recruitment of leukocytes that patrol the cerebral vasculature by crawling on the luminal endothelial surface [32], [33].

The mechanism responsible for detecting PTX and triggering the IL-6-mediated response described above is not elucidated. The theory holds that innate immune cells sense pathogen- and damage-associated molecules through a variety of membrane-bound and cytoplasmic receptors, such as those of the Toll-like and Nod-like families [34], [35]. Many of them engage signaling pathways that drive transcription of proinflammatory cytokines (e.g., IL-1β, TNF and IL-6). IL-1β differs from most of the others in that it is synthetized as an inactive form that must be cleaved to be activated and secreted. This cleavage is typically catalyzed by caspase-1 (Casp1), which is assembled into a mutiprotein complex called the inflammasome [36], [37]. The latter also includes a protein that serves as both a sensor for danger signals and a Casp1 activator. About ten inflammasome sensors have been characterized so far and most belong to the Nod-like receptor (NLR) family [38]. Whether such a sensor is involved in the response to PTX is not obvious *a priori*, since no increase in IL-1β is detectable in the plasma of PTX-treated mice [32].

In the present study, we sought to clarify how PTX is detected by the immune system and influences EAE development. We found that PTX induces IL-6 secretion from stromal cells indirectly via IL-1β, which is synthetized in myeloid cells under the control of the TLR4 pathway and the putative non-NLR inflammasome sensor pyrin (encoded by the *Mefv* gene). We also found that PTX requires pyrin to induce neutrophil adhesion to the cerebral endothelium of otherwise normal mice, and to effectively promote EAE in transgenic mice containing myelin-reactive T cells. Notably, these observations help us to appreciate the proinflammatory role of pyrin, which is currently a matter of debate, because divergent studies suggest that it either interacts with Casp1 through the adaptor protein ASC to form an active inflammasome [39]–[44] or acts as an inhibitor of Casp1 [45]–[48].

## Results

### PTX induces an inflammatory cascade involving myeloid cell-derived IL-1β and stromal cell-derived IL-6

**Figure 1:**
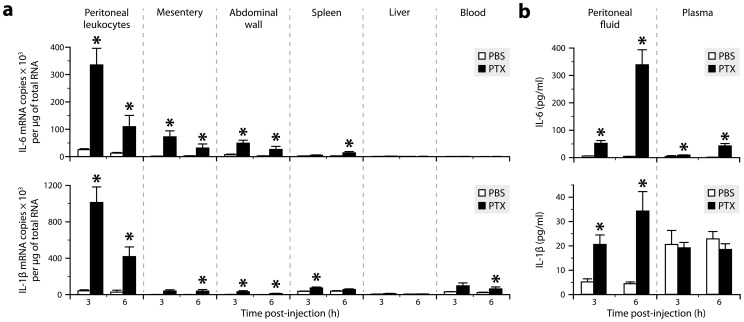
Both IL-6 and IL-1β are produced in tissues exposed to PTX, but only IL-6 reaches increased levels in the circulation. ***a***, Quantification of the mRNAs encoding IL-6 and IL-1β by qRT-PCR in different tissues from mice killed 3 or 6 h after intraperitoneal injection of PTX (20 µg/kg) or PBS. *Significantly different from the corresponding PBS group according to the Wilcoxon test (*P*<0.05). Sample size: 4–10 (PBS groups) or 7–10 (PTX groups). ***b***, Quantification of IL-6 and IL-1β by ELISA in peritoneal fluid and plasma samples. *Significantly different from the corresponding PBS group according to the Wilcoxon test (*P*≤0.0003). Sample size: 3–9 (PBS groups) or 4–10 (PTX groups).

We have reported that PTX promotes leukocyte adhesion to the brain vasculature by increasing the plasma level of IL-6 [32]. However, the molecular mechanism responsible for this upregulation and the cellular source of IL-6 remain unknown. To answer these questions, we first compared the IL-6 mRNA levels obtained by qRT-PCR in various tissues from mice killed 3 or 6 h after intraperitoneal injection of PTX (20 µg/kg). For comparison, we also measured the expression of IL-1β, a known regulator of IL-6 that does not increase in the blood of PTX-treated mice [32]. As shown in **Figure 1a**, PTX induced the transcription of both cytokines in peritoneal leukocytes and tissues surrounding the peritoneal cavity, including the mesentery and abdominal wall. To confirm these results at the protein level, we measured by ELISA the amounts of IL-6 and IL-1β in peritoneal fluid and blood samples collected from the same animals. As expected from our qRT-PCR data, and in agreement with our previous study [32], we found that both cytokines were secreted in the peritoneal fluid of PTX-treated mice, but that only IL-6 reached the circulation in significant amounts (**Fig. 1b**).

**Figure 2:**
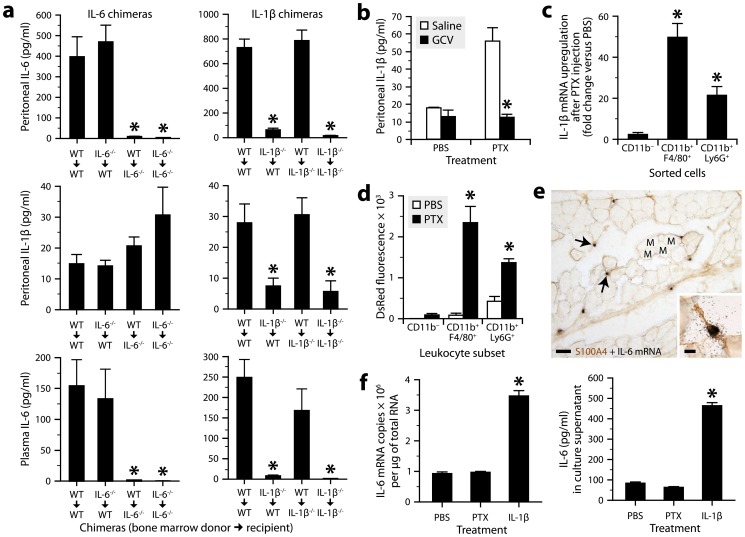
Radiosensitive peritoneal macrophages and neutrophils respond to PTX by producing IL-1β, which stimulates IL-6 production by radioresistant stromal cells. ***a***, Quantification of IL-1β and IL-6 by ELISA in peritoneal fluid or plasma collected from bone marrow chimeric mice 6 h after intraperitoneal injection of PTX (20 µg/kg) or PBS. *Significantly different from the controls (i.e., wild-type mice lethally irradiated and transplanted with bone marrow cells obtained from wild-type mice; WT→WT) according to *post hoc* Wilcoxon tests (Kruskal-Wallis test, *P*≤0.004). Sample size: 4–10 per group. ***b***, Quantification of IL-1β by ELISA in peritoneal fluid from CD11b-TK^mt-30^ mice treated twice daily for 6 days with GCV or saline, and killed 6 h after injection of PTX or PBS. *Significantly different from saline-treated mice injected with PTX according to the Wilcoxon test (*P* = 0.0017). Sample size: 4–7 per group. ***c***, Quantification of IL-1β mRNA by qRT-PCR in peritoneal leukocytes harvested from mice 6 h after injection of PTX or PBS, and sorted using the CD11b, F4/80 and Ly6G markers. *Significantly different from the corresponding PBS group according to the Wilcoxon test (*P*≤0.05). Sample size: 6–7 per group. ***d***, Comparison of DsRed fluorescence in macrophages (CD11b^+^F4/80^+^), neutrophils (CD11b^+^Ly6G^+^) and non-myeloid leukocytes (CD11b^−^) harvested from the peritoneum of pIL1-DsRed transgenic mice treated or not with PTX. Cells were analyzed by flow cytometry and gated on CD45. *Significantly different from the corresponding PBS group according to the Wilcoxon test (*P*≤0.008). Sample size: 5 (PBS groups) or 6 (PTX groups). ***e***, Micrographs showing *in situ* hybridization signals for IL-6 mRNA (clusters of black grains, arrows) in the abdominal wall of a mouse exposed for 3 h to PTX. The sections were counterstained for the mesenchymal/fibroblast marker S100A4 by immunohistochemistry (brown). Abbreviation: M, muscle bundle. Scale bars: 50 µm (main images) or 10 µm (insert). ***f***, Quantification of IL-6 expression by qRT-PCR or ELISA in primary cultures of S100A4^+^ abdominal stromal cells exposed for 3 h to PTX (100 ng/ml), IL-1β (10 ng/ml) or PBS. *Significantly different from the PBS group according to *post hoc* Wilcoxon tests (Kruskal-Wallis test, *P* = 0.0009). Sample size: 5–7 per group.

We next wanted to determine whether IL-1β mediates the effect of PTX on IL-6 expression and to identify the cell type(s) expressing these cytokines. We therefore generated chimeras by transplanting bone marrow cells into lethally irradiated mice using different combinations of donors and recipients expressing or lacking IL-1β or IL-6. In these mice, non-hematopoietic cells are radioresistant, while leukocytes are radiosensitive and replaced by donor-derived hematopoietic cells. ELISA on plasma and peritoneal fluid samples revealed that PTX could induce IL-6 secretion in wild-type mice reconstituted with IL-6-deficient bone marrow cells, but not in IL-6-deficient mice reconstituted with wild-type cells (**Fig. 2a**, left graphs). In contrast, PTX induced neither IL-6 nor IL-1β secretion in wild-type mice reconstituted with IL-1β-deficient bone marrow cells, but induced both cytokines in IL-1β-deficient mice reconstituted with wild-type cells (**Fig. 2a**, right graphs). These results indicate that IL-1β derives from radiosensitive leukocytes and regulates IL-6 expression in radioresistant cells.

We next performed a series of experiments to further pinpoint the cellular source of IL-1β and IL-6. First, we used CD11b-TK^mt-30^ transgenic mice expressing a mutated form of the *human herpesvirus 1 thymidine kinase* gene under the control of the myeloid-specific CD11b promoter [49]. In these mice, it is possible to deplete CD11b^+^ cells by the administration of ganciclovir (GCV), a prodrug that is converted by viral TK to a toxic nucleotide that kills proliferating cells. We found that CD11b-TK^mt-30^ mice responded normally to PTX by producing IL-1β, but not after myeloid cell depletion achieved through a 6-day treatment with GCV (**Fig. 2b** and **Fig. S1**). Second, we measured IL-1β mRNA in peritoneal leukocytes freshly harvested and purified by flow cytometry using the myeloid cell marker CD11b, the macrophage marker F4/80 and the neutrophil marker Ly6G. PTX upregulated IL-1β mRNA in CD11b^+^F4/80^+^ and CD11b^+^Ly6G^+^ leukocytes, but not in those negative for all these markers (**Fig. 2c**). Third, we injected PTX to transgenic mice expressing the fluorescent protein DsRed under the control of the IL-1β promoter [50], and analyzed their peritoneal leukocytes by flow cytometry. We observed that PTX increased DsRed expression almost exclusively in CD11b^+^F4/80^+^ and CD11b^+^Ly6G^+^ leukocytes (**Fig. 2d** and **Fig. S2**). Fourth, knowing that IL-6 derives from radioresistant cells, abdominal wall sections from PTX-treated mice were analyzed for the presence of IL-6 mRNA by radioisotopic *in situ* hybridization and immunostained or not for the mesenchymal/fibroblastic marker S100A4 (also called fibroblast-specific protein-1). Strong hybridization signals were detected in the connective tissue surrounding the muscle bundles (**Fig. 2e**, main image), where they colocalized with S100A4 immunostaining (**Fig. 2e**, insert). No signal was detected in sections from PBS-treated mice (data not shown). Finally, we used mouse abdominal wall biopsies to prepare primary cultures of S100A4^+^ stromal cells. The aim was to determine whether these cells had the ability to produce IL-6 when exposed for 3 h to PTX or IL-1β. The results showed a strong upregulation of IL-6 at the mRNA and protein levels in the presence of IL-1β, but not of PTX (**Fig. 2f**). Overall, these results demonstrate that PTX stimulates peritoneal macrophages and neutrophils to produce IL-1β, which in turn stimulates IL-6 synthesis in abdominal stromal cells.

### PTX induces the IL-1β−IL-6 cascade through the TLR4 pathway and pyrin inflammasome in a manner dependent on its ADP-ribosyltransferase activity

**Figure 3:**
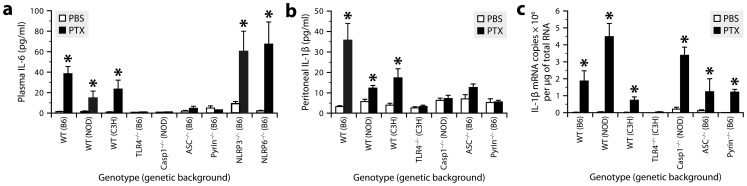
PTX induces the IL-1β−IL-6 cascade through the TLR4 pathway and pyrin inflammasome. ***a***, Quantification of IL-6 by ELISA in plasma from mice of different genotypes killed 6 h after intraperitoneal injection of PTX (20 µg/kg) or PBS. *Significantly different from the corresponding PBS group according to Wilcoxon tests (*P*≤0.0066). Sample size: 4–17 (PBS groups) or 7–15 (PTX groups). ***b***, Quantification of IL-1β by ELISA in peritoneal fluid from mice treated or not with PTX. *Significantly different from the corresponding PBS group according to Wilcoxon tests (*P*≤0.017). ***c***, Quantification of IL-1β mRNA by qRT-PCR in peritoneal leukocytes from mice treated or not with PTX. *Significantly different from the corresponding PBS group according to Wilcoxon tests (*P*≤0.022).

To clarify the immune recognition mechanism through which PTX induces the IL-1β−IL-6 cascade, we examined mice lacking either TLR4 or inflammasome components for a defect in IL-6 expression after intraperitoneal injection of PTX. ELISA results revealed a normal increase of plasma IL-6 after PTX injection in mice lacking the ASC-dependent inflammasome sensors NLRP3 and NLRP6, but no increase in mice lacking TLR4, Casp1, ASC or pyrin (**Fig. 3a** and **Fig. S3**). We next further examined the latter mice for a defect in IL-1β synthesis. Consistently, none of them had an increased amount of IL-1β in their peritoneal fluid (**Fig. 3b**), despite the fact that those lacking Casp1, ASC or pyrin exhibited a normal increase of IL-1β mRNA in their peritoneal leukocytes (**Fig. 3c**). In contrast to PTX, the TLR4 agonist lipopolysaccharide (LPS) could induce IL-1β and IL-6 secretion in pyrin-deficient mice (**Fig. S4**), indicating that the absence of pyrin causes a stimulus-specific defect in IL-1β production. Altogether, these results support the concept that PTX-driven inflammation operates under multiple levels of control; it induces IL-1β transcription through the TLR4 signaling pathway and then requires function of the pyrin inflammasome to induce IL-1β post-translational processing.

**Figure 4:**
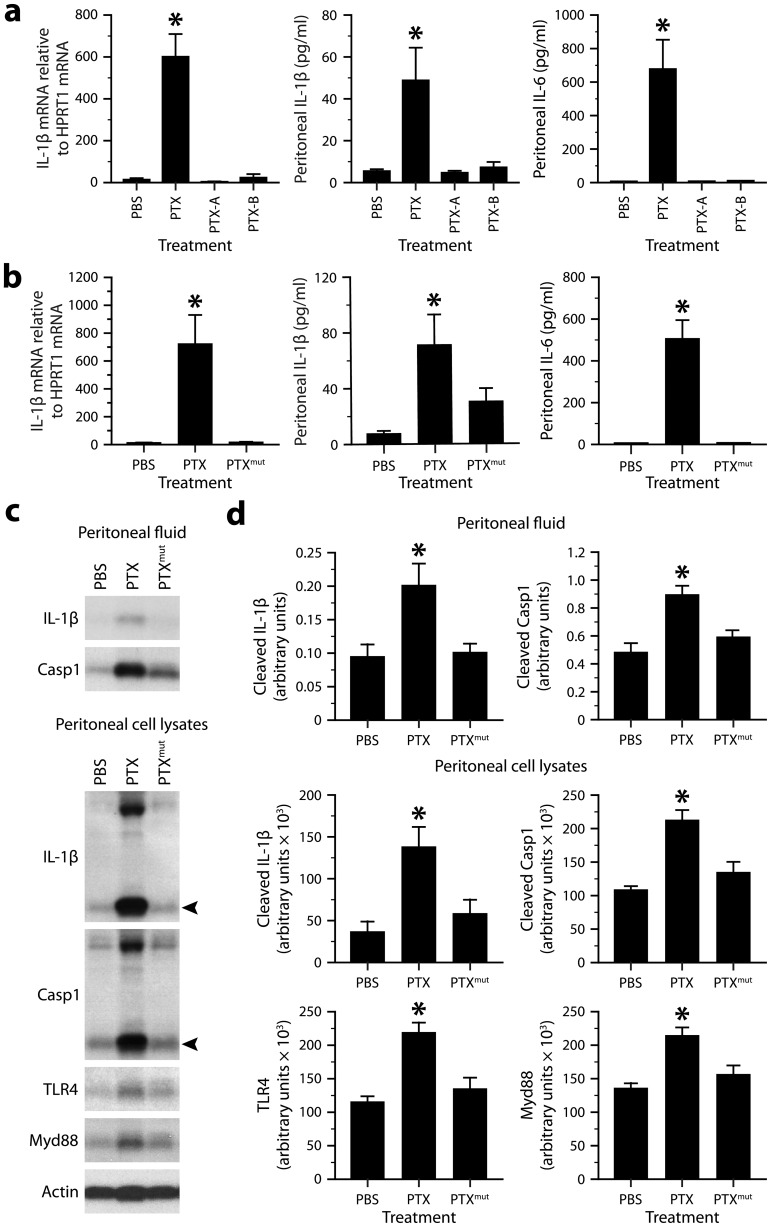
The ability of PTX to induce the IL-1β−IL-6 cascade depends on the integrity of its multimeric structure and enzymatic activity. ***a***, Quantification of IL-1β mRNA in peritoneal leukocytes by qRT-PCR, and of IL-1β and IL-6 protein in peritoneal fluid by ELISA. The samples were from mice killed 6 h after intraperitoneal injection PBS or equimolar doses of PTX (20 µg/kg), PTX-A (5.3 µg/kg) or PTX-B (14.5 µg/kg). *Significantly different from all the other groups according to *post hoc* Wilcoxon tests (Kruskal-Wallis test, *P*≤0.001). Sample size: 16 (PBS group) or 8–11 (toxin groups). ***b***, Same analyzes as in *a*, except that mice received PTX^mut^ (20 µg/kg). *Significantly different from all the other groups according to *post hoc* Wilcoxon tests (Kruskal-Wallis test, *P*≤0.0012). Sample size: 10–12 per group. ***c***, Western blots revealing the presence of mature IL-1β, cleaved Casp1, TLR4 and Myd88 in peritoneal fluid or peritoneal cell lysates from mice killed 6 h after injection of PBS, wild-type PTX or PTX^mut^. Arrowheads indicate the cleaved forms. β-actin was used as loading control. ***d***, Quantification of the Western blots by optical densitometry. *Significantly different from all the other groups according to *post hoc* Wilcoxon tests (Kruskal-Wallis test, *P*≤0.02). Sample size: 8 per group.

To test whether the ability of PTX to induce the IL-1β−IL-6 cascade depends on the integrity of its multimeric structure and ADP-ribosyltransferase activity, we compared the expression levels of these cytokines in mice exposed to different forms of PTX, including the A protomer (PTX-A) and B oligomer (PTX-B) separately, and an enzymatically inactivated mutant (PTX^mut^) with 2 amino acid substitutions (R9K and E129A). The latter has an antigenic structure comparable to that of the wild-type form, but an ADP-ribosyltransferase activity below 0.01% [51]. We found that all these forms, except the wild-type one, were unable to activate IL-1β transcription in peritoneal leukocytes and secretion of IL-1β and IL-6 in the peritoneal fluid (**Fig. 4a, b**). Western blots confirmed and extended these results by showing increased levels of cleaved Casp1, mature IL-1β, TLR4 and MyD88 only in mice challenged with wild-type PTX (**Fig. 4c, d**). These observations suggest that PTX depends on both its multimeric structure and its ADP-ribosyltransferase activity to induce IL-1β production.

Because PTX strongly induces neutrophil recruitment into the peritoneum (**Fig. S1** and **S2**), we asked whether the reduced expression of cytokines observed in pyrin-knockout mice could be attributable to a change in the number of infiltrating leukocytes. We therefore analyzed peritoneal leukocytes from pyrin-knockout and wild-type mice by flow cytometry using various leukocytic markers. The most noticeable effect of PTX was a marked increase in the number of CD11b^+^Ly6G^+^ neutrophils, but no intergenotype difference was recorded (**Fig. S5**). These results indicate not only that the differences in cytokine expression were not the consequence of a difference in the cellular composition, but also that neutrophil infiltration into the peritoneum in response to PTX is neither dependent on pyrin nor the IL-1β−IL-6 cascade.

**Figure 5:**
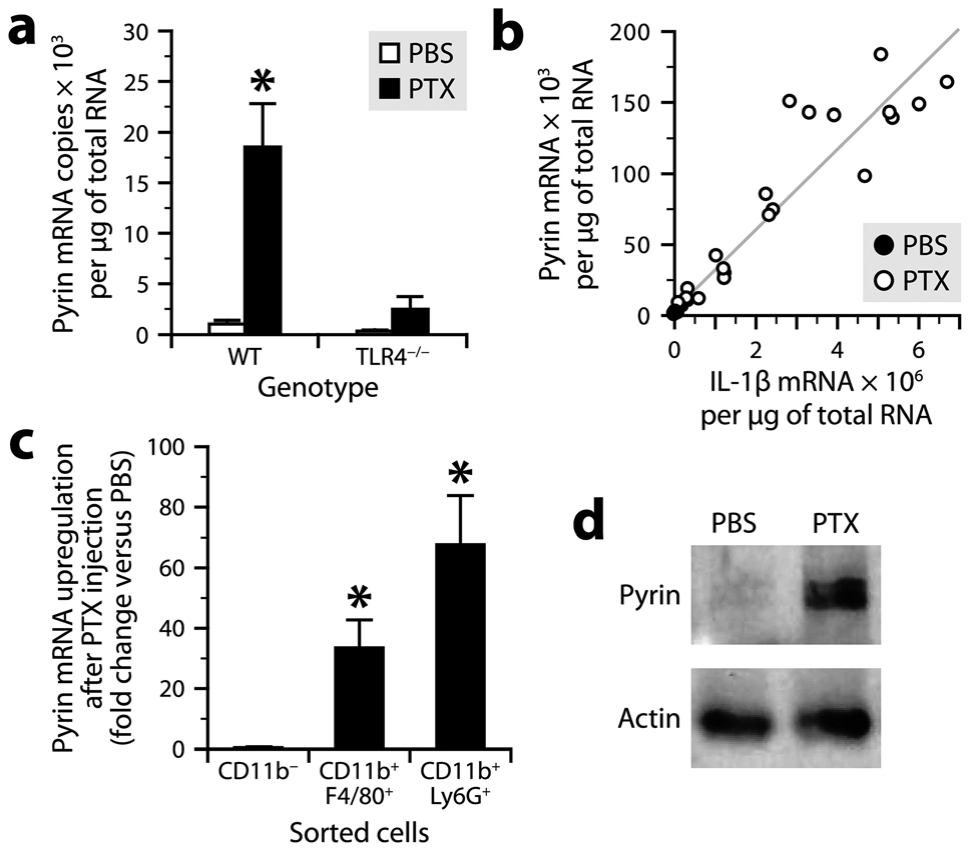
PTX increases pyrin expression in myeloid cells via a TLR4-dependent pathway. ***a***, Quantification of pyrin mRNA by qRT-PCR in peritoneal leukocytes from TLR4-knockout and wild-type mice harvested 6 h after intraperitoneal injection of PTX (20 µg/kg) or PBS. *Significantly different from all the other groups according to Wilcoxon tests (*P*≤0.014). Sample size: 8 (PTX groups) or 4 (PBS groups). ***b***, Bivariate analysis showing a positive correlation between the amounts of pyrin and IL-1β mRNAs (Spearman's test, *P*<0.0001, R = 0.98). ***c***, Quantification of pyrin mRNA by qRT-PCR in peritoneal leukocytes harvested from mice 6 h after injection of PTX or PBS, and sorted using the CD11b, F4/80 and Ly6G markers. *Significantly different from the corresponding PBS group according to the Wilcoxon test (*P*≤0.0081). Sample size: 6–7 per group. ***d***, Pyrin detection by Western blotting in peritoneal cells from mice killed 6 h after injection of PTX or PBS. β-actin was used as loading control.

Finally, we asked whether the *in vivo* effect of PTX on IL-1β production could be reproduced *ex vivo* by using freshly isolated peritoneal leukocytes, which were stimulated for 6 h at 37°C. ELISA results showed that the amount of IL-1β was strongly increased in cell extracts after treatment with PTX (20 µg/ml), but weakly with heat-inactivated PTX and not at all with PTX^mut^ (**Fig. S6a**). However, only a very small increase in IL-1β was detected in the supernatant of cells treated with PTX. Similar results were obtained using pyrin-deficient leukocytes (**Fig. S6b**). The deficient secretion of IL-1β from wild-type leukocytes was not due to an absence of pyrin expression, which was normally increased in response to PTX and correlated with IL-1β expression (**Fig. S6c**), as previously observed *in vivo* (**Fig. 5b**). These observations suggest that PTX can actually trigger IL-1β and pyrin synthesis *ex vivo*, but not pyrin activation, which is required for IL-1β processing and release and which depends on more complex conditions found *in vivo*.

### PTX induces pyrin expression in myeloid cells via TLR4

To determine whether and how PTX regulates pyrin expression, we performed qRT-PCR on mRNA from peritoneal leukocytes of TLR4-knockout and wild-type mice harvested 6 h after exposure or not to PTX. The results showed that the levels of pyrin mRNA were low in non-stimulated leukocytes, but 18 times higher in PTX-exposed leukocytes (**Fig. 5a**), in which they positively correlated with the levels of IL-1β mRNA (**Fig. 5b**). However, this upregulation was not observed in TLR4-deficient leukocytes (**Fig. 5a**). Consistent with the results obtained for IL-1β (**Fig. 4b**), a repetition of the experiment using PTX^mut^ revealed no change in pyrin mRNA (303±151 copies versus 365±145 for the PBS group and 1722±221 for the PTX group). To determine the cellular source of pyrin, we measured its mRNA by qRT-PCR in sorted peritoneal cells. We found that PTX increased pyrin mRNA expression in CD11b^+^F4/80^+^ and CD11b^+^Ly6G^+^ leukocytes, but not in the CD11b^−^ population (**Fig. 5c**). Finally, to confirm the inducible nature of pyrin at the protein level, we analyzed peritoneal cell lysates by Western blotting using a polyclonal antibody directed against the N-terminal 410 amino acids of mouse pyrin and recognizing two major splicing variants [45]. A band doublet at the expected molecular weight of ∼100 kDa was detected in peritoneal cell lysates from mice treated with PTX, but not from control mice (**Fig. 5d**). We conclude from these results that the ADP-ribosyltransferase activity of PTX leads to *de novo* expression of pyrin in macrophages and neutrophils via a TLR4-dependent mechanism.

### Pyrin contributes to PTX-induced neutrophil intravascular patrolling and EAE

The CNS microvasculature is constantly patrolled by rod-shaped leukocytes that crawl on the luminal endothelial surface [52], [53]. This cell population is mainly composed of monocytes and neutrophils under normal conditions and within a few hours after exposure to bacterial toxins, but also includes a significant proportion of lymphocytes during EAE [32]. We have shown that PTX increases the number of crawling neutrophils in brain capillaries through an IL-6-dependent mechanism, but we have failed to demonstrate a similar effect on lymphocytes, at least during the first 6 h after PTX injection [32]. To examine the possibility that such an increase occurs after a longer period, we administered PTX to mice according to a protocol commonly used for EAE induction (i.e., 2 intraperitoneal injections of 20 µg/kg given 2 days apart). Twenty-four hours after the last injection, the brains were perfused to remove non-adherent leukocytes and collected to assess the presence of rod-shaped CD3^+^ T cells in the capillary network of the cerebral cortex. Stereological analysis revealed a 3.5-fold increase of these cells in PTX-treated mice, reaching a density of approximately 141 cells per mm^3^ compared to 40 in control mice (*n* = 3 per group). As expected, the effect was not limited to lymphocytes, because a concomitant 4.2-fold increase of rod-shaped Ly6G^+^ neutrophils was observed (139 cells per mm^3^ versus 33 in controls).

**Figure 6:**
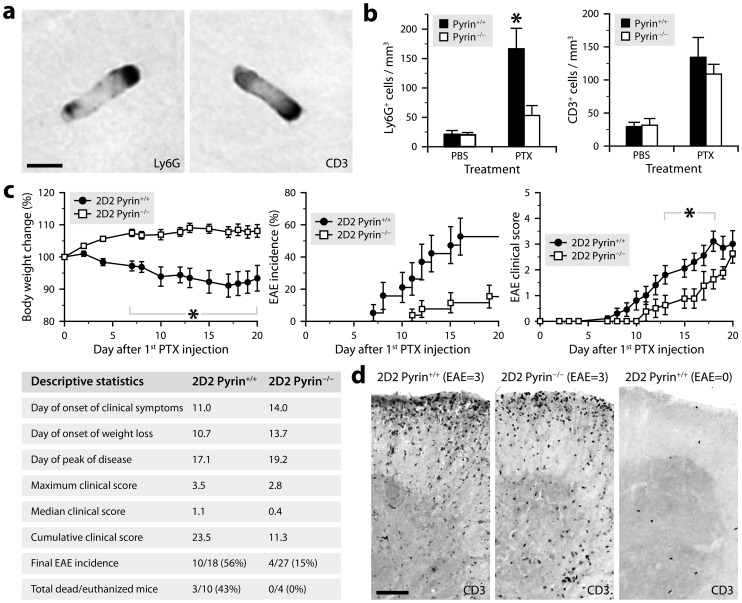
PTX requires pyrin to induce neutrophil intravascular crawling and to maximally promote EAE. ***a***, Micrographs showing rod-shaped leukocytes stained for Ly6G or CD3 by immunohistochemistry in brain sections from a PTX-treated mouse (C57BL/6 background). Scale bar: 5 µm. ***b***, Stereological counts of rod-shaped Ly6G^+^ and CD3^+^ leukocytes in the cerebral cortex of C57BL/6 mice deficient (white bars) or not (black bars) in pyrin and killed 24 h after treatment with PTX (2 injections of 20 µg/kg given 2 days apart) or PBS. *Significantly different from the corresponding pyrin-deficient group according to the Wilcoxon test (*P* = 0.0050). Sample size: 6–7 (PBS groups) or 7–10 (PTX groups). ***c***, Monitoring of EAE in PTX-treated 2D2 mice expressing (black circles) or lacking pyrin (white squares). All mice were included in the analyses, except for the clinical scoring (right graph), which included only mice that had developed clinical signs of EAE at the end of the study (i.e., after 21 days). *Time at which a significant intergenotype difference was found according to Wilcoxon tests (*P*≤0.044). The Kaplan-Meier curves (EAE incidence) were significantly different according to the Wilcoxon test (*P* = 0.013). Additional statistics are provided in the table. Sample size: 12 (2D2 Pyrin^+/+^) or 19 (2D2 Pyrin^−/−^). ***d***, Micrographs of spinal cord sections showing comparable infiltration of CD3^+^ T cells in 2D2 mice expressing or not pyrin with an EAE score of 3, but few cells in a mouse that did not develop EAE. These mice were killed 21 days after the first PTX injection. Scale bar: 100 µm.

**Figure 7:**
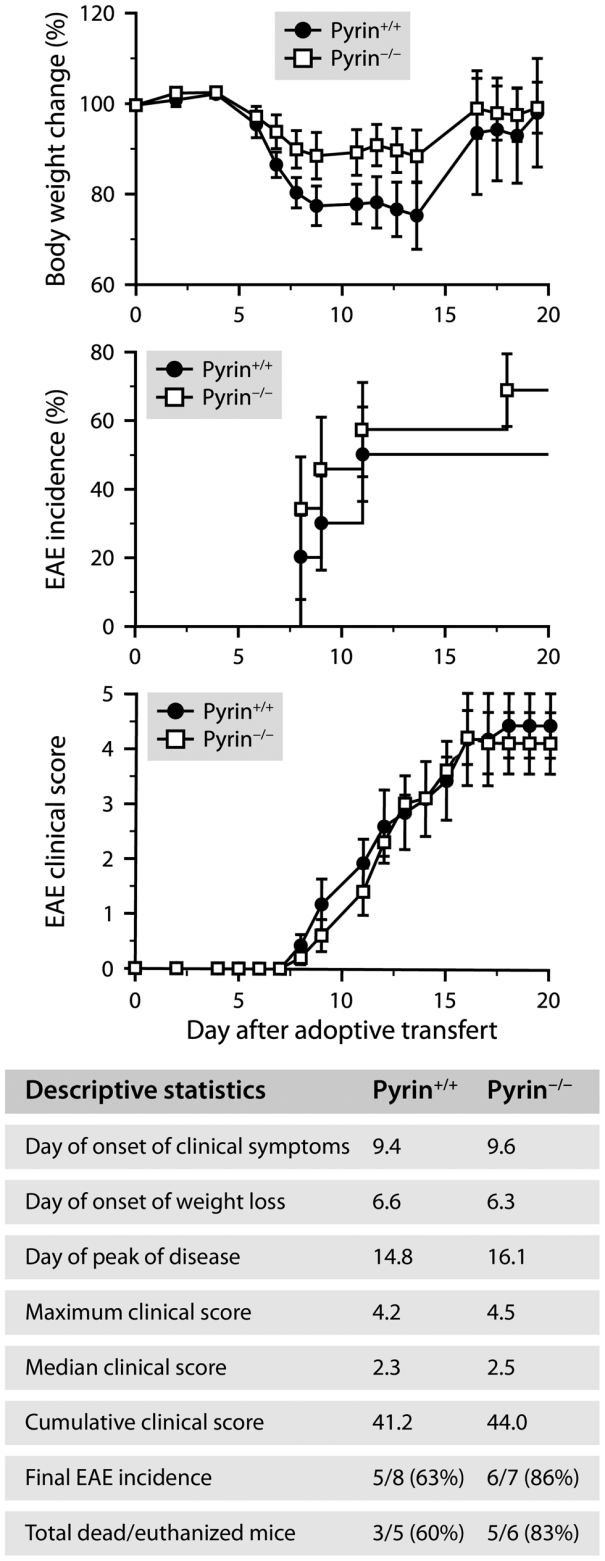
Pyrin is not required for the development of EAE induced by adoptive transfer of encephalitogenic T cells. Monitoring of EAE in C57BL/6 mice expressing (black circles) or lacking pyrin (white squares) after adoptive transfer of lymphocytes isolated from MOG-immunized wild-type mice and reactivated *in vitro*. All mice were included in the analyses, except for the clinical scoring (bottom graph), which included only mice that had developed clinical signs of EAE at the end of the study (i.e., after 21 days). No significant intergenotype difference was detected according to Wilcoxon tests (*P*≥0.2). Additional statistics are provided in the table. Sample size: 8 (Pyrin^+/+^) or 7 (Pyrin^−/−^).

Having found that the regimen of PTX used to promote EAE increases intravascular patrolling by both lymphocytes and neutrophils, we wondered whether pyrin: 1) is involved in the underlying mechanism; and 2) contributes to the effect of PTX on EAE, that is, a CD4^+^ T cell-mediated disease in which neutrophils play an important role [33], [54]–[56]. To answer these questions, we used pyrin-deficient mice in a C57BL/6 or 2D2 background. 2D2 mice express a transgenic myelin oligodendrocyte glycoprotein (MOG)-specific T cell receptor in most of their CD4^+^ lymphocytes and can develop EAE at a reported incidence of 39% if exposed to PTX under specific pathogen-free housing conditions [57]. First, we found that pyrin-deficient C57BL/6 mice exhibited no increase in the recruitment of rod-shaped Ly6G^+^ neutrophils on the cerebral vasculature after treatment with PTX, contrary to wild-type controls and despite a normal increase in the number of adherent CD3^+^ lymphocytes (**Fig. 6a,b**). Second, the cohort of pyrin-deficient 2D2 mice developed EAE in response to PTX at an incidence ∼3 times lower compared to pyrin-expressing 2D2 mice (**Fig. 6c**). The pyrin-deficient 2D2 mice that developed EAE did it with a 4-day delay and with less severity during the progressive phase of the disease (**Fig. 6c**, right graph). However, comparable infiltration of CD3^+^ cells was observed in the spinal cord of mice with similar clinical scores killed after 21 days, regardless of their genotype at the *Mefv* locus (**Fig. 6d**). This suggested that pyrin might not be essential for the effector phase of EAE, when myelin-reactive lymphocytes have been activated and recruited to the CNS parenchyma. This hypothesis was confirmed by the observation that pyrin-deficient and wild-type mice developed similar EAE when adoptively transferred with encephalitogenic T cells isolated from MOG-immunized wild-type mice and reactivated *in vitro* (**Fig. 7**). Altogether, these results demonstrate that pyrin contributes to two different, but potentially related phenomena: the induction of neutrophil adhesion to CNS capillaries and the activation of the initial phase of EAE by PTX.

## Discussion

This study provides answers to the long-lasting question of how PTX produces an adjuvant effect that promotes EAE. First, contrary to the prevailing concept [30], our results suggest that PTX does not act directly via membrane-bound receptors such as TLR4, but rather by catalyzing the ADP-ribosylation of G-protein α_i_ subunits, a phenomenon that requires the internalization and disassembling of PTX to release its A protomer in the cytoplasm. Indeed, mutant PTX, containing only 2 amino acid substitutions in one of its 6 subunits, was unable to activate IL-1β transcription (Fig. 4), which was dependent on TLR4 (Fig. 3). The mutations affect the enzymatic activity of the A-protomer, but not the antigenic structure of the whole toxin [51]. The protein binding properties of mutant PTX are thus likely to be comparable to that of wild-type PTX. Therefore, we propose that PTX causes perturbations in G protein-coupled receptor signaling (e.g., cAMP-dependent pathways), leading to the release of an endogenous danger signal called alarmin that activates TLR4 on myeloid cells and drives IL-1β transcription. Several TLR4-interacting alarmins have been identified so far, including HMGB1, S100A8/A9 and heat-shock proteins [58]. Additional work will be needed to further clarify the mechanism by which TLR4 is activated in response to PTX. Meanwhile, we speculate that genetic defects or other factors that may disrupt G protein-coupled receptor signaling in the manner of PTX might contribute to MS and MS-like diseases.

A second answer is that PTX promotes the formation of a pyrin-dependent inflammasome that cleaves pro-IL-1β into its active form (and perhaps other Casp1-dependent cytokines such as IL-18). To our knowledge, PTX represents the first microbial molecule known to activate pyrin. Our observations are in agreement with the view that pyrin exerts a proinflammatory function, as supported by studies showing that pyrin interacts with ASC and activates Casp1 [39]–[44]. Mutations in the gene encoding pyrin (*MFEV*) are known to cause familial Mediterranean fever [59]–[61], a hereditary inflammatory disorder characterized by recurrent fevers and painful inflammation. Interestingly, some of these mutations have been linked to a higher susceptibility of developing a more progressive or severe form of MS [62]–[65]. The question of whether defects in inflammasome components such as pyrin could reduce the activation threshold of autoreactive T cells should fuel future investigations.

A third answer may reside in the systemic innate immune response triggered by IL-1β. Indeed, after being secreted by myeloid cells exposed to PTX, IL-1β stimulates nearby stromal cells to produce IL-6, which passes into the bloodstream. As previously shown [32], [33], IL-6 induces the expression of ICAM1 and CXCR2 ligands at the surface of cerebral endothelial cells, leading to the adhesion and crawling of neutrophils through interaction between CD11b and ICAM1. The question arises as to whether neutrophils mediate the effect of PTX on EAE. Mounting evidence actually suggests a major involvement of neutrophils in this model. It has been reported that neutrophils massively infiltrate the CNS of EAE mice [32], [54], [66]–[73], and that EAE is attenuated in mice lacking the neutrophil receptor CXCR2 or treated with antibodies against CXCR2, its ligand CXCL1 or the neutrophil-specific protein Ly6G [33], [54], [56], [74], [75]. Although their functions in EAE are unknown, it is hypothesized that neutrophils may initiate the disease by altering the permeability of the blood-brain barrier, thereby allowing access to other immune cells [55]. It is also proposed that neutrophils secrete proinflammatory molecules that promote the maturation of monocytic cells into professional antigen-presenting cells [56], which are essential for local restimulation of myelin-reactive T cells. In human, neutrophils play a role in neuromyelitis optica [76], an autoimmune disorder that resembles MS in some ways, but the possibility that they contribute to MS is questionable. While neutrophils have not been traditionally observed in increased number in fluids and postmortem tissues from MS patients, more recent studies show that neutrophils with an activated phenotype are more numerous in the blood of MS patients and that the CXCR2 ligand IL-8 is elevated [77], [78].

Of course, it is also possible that the IL-1β−IL-6 cascade exerts neutrophil-independent effects, such as the bystander activation of autoreactive T lymphocytes, defined as a process that does not require specific TCR stimulation (e.g., it can occur via soluble mediators or cross-linking of membrane-bound receptors such as CD2 and CD28 [79]). IL-6 is a multifunctional proinflammatory cytokine that is essential in the development of autoimmune diseases, including EAE [80]–[85]. It is believed to act by stimulating the generation of autoreactive Th17 lymphocytes in the presence of TGFβ [86]–[89]. IL-6 and IL-17 have been shown to be engaged in a positive feedback loop that sustains inflammation and contributes to autoimmune responses [85], [90], [91]. However, IL-6 is not sufficient in itself and most likely requires another factor, because CD3+ cells increase normally in number in the CNS vasculature of pyrin-KO mice challenged with PTX, despite a lack of IL-6 expression. This observation suggests the existence of another PTX-induced pathway that regulates lymphocyte trafficking independently of pyrin, IL-1β and IL-6. This pathway could also be involved in the PTX-induced recruitment of neutrophils into the peritoneum, which is independent on pyrin, contrary to the recruitment of crawling neutrophils at the surface of the CNS vasculature.

The effects of PTX are paradoxical: how can a chemotaxis-blocking agent induce leukocyte recruitment? A possible explanation is that these effects are mediated by distinct mechanisms that are separated in space and time. Indeed, early after injection, PTX is likely to be internalized by different cells, including peritoneal leukocytes, whose migratory ability would be blocked, as previously documented [26]. A few hours later, affected cells would release an alarm signal that would trigger pyrin-dependent and independent mechanisms, leading to the recruitment of neutrophils into the peritoneum and of crawling leukocytes onto the luminal surface of the systemic vasculature. The recruitment of these cells would not be blocked by PTX, because it would occur at a distant site or when PTX is likely to have been cleared from the extracellular peritoneal milieu (e.g., by cellular internalization and passive diffusion).

An important question to address in future work is how PTX activates pyrin. This protein comprises: 1) a N-terminal pyrin domain that binds to ASC [92], an adaptor that links pyrin to Casp1; 2) a B-box type zinc finger domain that interacts with other proteins (e.g., mutant PSTPIP1 [41], [93]); and 3) a coiled-coil domain responsible for homotrimerization of pyrin [41]. While the C-terminal region of mouse pyrin is uncharacterized, the human counterpart contains a SPRY domain that mediates interactions with Casp1 and pro-IL-1β and that is the site of mutations associated with Mediterranean fever [94]. It has been reported that the binding of mutant PSTPIP1 to the B-box activates pyrin by causing a conformational change that releases the pyrin domain from sequestration, thus promoting the recruitment of the ASC-Casp1 complex [41]. Similarly, it is tempting to speculate that pyrin would act as a sensor for an endogenous danger signal inducible by PTX *in vivo* (but not *ex vivo*), which would mimic the effect of mutant PSTPIP1 by binding to the B-box.

In conclusion, this study provides fundamental insights into how the immune system recognizes PTX. By analogy, it helps to appreciate how microbial agents may influence the course of autoimmune diseases, such MS, which is worsened by common infections. By identifying PTX as a pyrin activator as well as an inducer of lymphocyte crawling, this study provides a new convenient model for investigating the mechanisms that control the activation of the pyrin inflammasome and the trafficking of lymphocytes at the blood-brain interface. This study also provides the first physiological demonstration of a role of wild-type pyrin *in vivo*, as opposed to the mutated forms found in patients suffering from Mediterranean fever. Finally, it identifies a pyrin-dependent innate immune response that could potentially be targeted for the treatment of autoimmune diseases.

## Materials and Methods

### Ethics statement

All mice experiments were approved by the Laval University Animal Protection Committee (permit #2012140) and were conducted in compliance with the guidelines of the Canadian Council on Animal Care.

### Mice

The mice used in this study are detailed in **Table S1**. They were acclimated to our facility for at least 1 week, housed under specific pathogen-free conditions and tested at 8–10 weeks of age.

### Toxin injection

Mice were injected intraperitoneally with PTX (20 µg/kg), PTX-A (5.3 µg/kg), PTX-B (14.5 µg/kg), PTX^mut^ (20 µg/kg), LPS from *Escherichia coli* O55:B5 (1 mg/kg) or PBS. The toxins were purchased from List Biological Laboratories, except LPS (Sigma-Aldrich). In most experiments, mice were given a single injection and killed 3 or 6 h later. In indicated experiments, they were given 2 injections, 2 days apart, and killed 24 h after the last injection.

### Ganciclovir treatment

Mice were injected intraperitoneally twice daily for 6 days with GCV (Hoffmann-La Roche, 50 mg/kg, diluted in saline). Control mice were treated identically, except that GCV was substituted by saline.

### Bone marrow transplantation

Recipient mice were exposed to 10 gray of radiation using a ^137^caesium source (Gammacell 40 Exactor) and injected via a tail vein with 10^7^ bone marrow cells freshly collected from donor mice. The cells were harvested by flushing femurs with DPBS containing 2% FBS using a syringe with a 25-gauge needle, filtered through a 40-µm nylon mesh, centrifuged and resuspended in DPBS. The mice were treated with antibiotics (Septra; 100 µg/ml trimethoprim and 500 µg/ml sulfamethoxazole in drinking water) for 3 days before and 3 weeks after transplantation. Chimeric mice were injected with PTX 2 months after transplantation.

### EAE induction by active immunization

Mice were subcutaneously injected into both flanks with a total of 200 µl of emulsion containing 300 µg of MOG peptide 35–55 (Feldan) dissolved in saline and mixed with an equal volume of complete Freund's adjuvant containing 500 µg of killed *Mycobacterium tuberculosis* H37 RA (Difco Laboratories). The mice were also injected intraperitoneally with 20 µg/kg of PTX immediately and 2 days after immunization.

### EAE induction by adoptive transfer

Mice were intraperitoneally injected with 20×10^6^ lymphoid cells. These were isolated from abdominal lymph nodes and spleens of mice killed 8 days after active EAE induction, and then cultured for 2 days in DMEM supplemented with MOG peptide 35–55 (15 µg/ml), murine IL-12 (5 ng/ml), murine IL-23 (20 ng/ml), FBS (10%), 1% modified Eagle's medium nonessential amino acids (Wisent), penicillin (100 U/ml), streptomycin (100 µg/ml) and amphotericin B (250 ng/ml).

### Evaluation of EAE symptoms

Mice were weighed and scored daily as follows: 0, no visual sign of disease; 0.5, partial tail paralysis; 1, complete tail paralysis; 1.5, weakness in one hind limb; 2, weakness in both hind limbs; 2.5, partial hind limb paralysis; 3, complete hind limb paralysis; 3.5, partial forelimb paralysis; 4, complete forelimb paralysis; 5, dead or killed for humane reasons.

### Cell culture

Primary cultures of abdominal stromal cells were prepared from peritoneal wall biopsies and mesentery of adult mice. The tissues were washed in PBS and digested in 0.25% trypsin-EDTA solution for 50 min at 37°C with gentle agitation. The mixture was filtered on a 70-µm cell strainer and centrifuged at 1,500 rpm for 5 min. Cells were cultured in DMEM supplemented with 15% heat-inactivated FBS, penicillin (100 U/ml), streptomycin (100 µg/ml) and amphotericin B (250 ng/ml) until confluence. Cells were subcultured in DMEM containing 10% FBS and used at passage 3. For experimentation, 1.5×10^5^ cells were seeded in 6-well plates, starved for 24 h and incubated for 3 h with 100 µg/ml PTX, 10 ng/ml recombinant mouse IL-1β (BioLegend) or PBS as control.

### *Ex vivo* stimulation of leukocytes

Peritoneal leukocytes were harvested by flushing the peritoneum of naive mice with 5 ml D-PBS. Cells were pooled in DMEM supplemented with 2% FBS, transferred in 24-well plates at a density of 2×10^6^ cells per well in 500 µl containing or not 500 ng/ml PTX, PTXmut or heat-inactivated PTX (heated at 80°C for 30 min). After a 6 h-incubation at 37°C, the supernatant was collected, and the cells were washed with PBS and then lyzed with Cell Lysis Buffer 2 (R&D Systems).

### RNA isolation and qRT-PCR

Total RNA was isolated from tissue or cells by homogenization in TRI-reagent (Sigma-Aldrich) followed by purification using the GenElute Mammalian Total RNA Miniprep Kit (Sigma-Aldrich). First strand cDNA was generated from 1 (cells) or 5 (tissues) µg of total RNA using Superscript III (Invitrogen) with random hexamers and 20-mer oligo-dT primers, then purified using the GenElute PCR Clean-Up Kit (Sigma-Aldrich). The product (20 ng) was analyzed using the LightCycler 480 system with the SYBR Green I Master mix according to the manufacturer's instructions (Applied Biosystems). The primers were as follows: IL-1β, 5′-TCAAATCTCGCAGCAGCACATC-3′ and 5′- CCAGCAGGTTATCATCATCATCCC-3′; IL-6, 5′-GTCCTTCCTACCCCAATTTCCAA-3′ and 5′- GAATGTCCACAAACTGATATGCTTAGG-3′; pyrin, 5′-CCCTACTGGATGAGATGATTGAAGAAC-3′ and 5′-TCCAACAGCTCAGAGGCAGACAT-3′. The PCR conditions consisted of 45 cycles of denaturation (10 s at 95°C), annealing (10 s at 60°C), elongation (14 s at 72°C) and reading (5 s at 74°C). The number of mRNA copies was determined using the second derivative method [95].

### ELISA

Blood samples were obtained by intracardiac puncture in EDTA tubes and centrifuged for 20 min at 2000×g to collect plasma. Peritoneal fluid was collected by washing the peritoneal cavity with 2 ml of DPBS, and then centrifuged for 3 min at 1000×g to remove peritoneal cells. Samples were analyzed using the mouse IL-1β Quantikine ELISA kit or IL-6 Duoset ELISA kit (R&D Systems).

### Western blotting

Peritoneal fluid and cell samples were homogenized in extraction buffer (20 mM Tris-HCl at pH 7.5, 150 mM NaCl, 1% Triton X-100, 1 mM ethylenediaminetetraacetic acid, 1 mM ethylene glycol tetraacetic acid, 2.5 mM pyrophosphate, 1 mM β-glycerophosphate, 1× protease inhibitor cocktail [Sigma]). Immunoblot analysis was carried out with the Criterion system (Bio-Rad). Accordingly, proteins were resolved in 4–20% Tris-HCl Criterion precasted gels (Bio-Rad), transferred to polyvinylidene difluoride membranes (Applied Biosystems), placed in blocking buffer (PBS, 0.1% Tween 20 and 0.4% I-Block [Applied Biosystems]), and then incubated for 1 h with antibodies to IL-1β (1∶5000, National Cancer Institute), Casp1 (1∶1000, Imgenex), TLR4 (1∶1000, Imgenex), Myd88 (1∶1000, Imgenex) or pyrin (1∶10000, Dr. Jae Jin Chae), followed by 45 min in appropriate secondary horseradish peroxidase (HRP)-linked antibodies (Cell Signaling Technology). Signal visualization was enhanced by chemiluminescence using a phototope-HRP detection kit (Cell Signaling Technology). In cell lysates, to control for protein loading, immunoblots were stripped with Restore Western blot stripping buffer (Pierce) and blotted for β-actin using monoclonal anti-β-actin antibody (1∶5000, Sigma). Data were normalized to β-actin for peritoneal cells and equal amounts of protein were loaded for peritoneal fluid (20 µg). Band densities were quantified with UN-SCAN-IT software 5.3, measuring the gray scale intensity, subtracting single region from the background value, checking for saturation and with a linear optical density calculation.

### Flow cytometry

Peritoneal cells were blocked for 10 min with 5 µg/ml anti-CD16/CD32 antibody (clone 2.4G2, BD Biosciences) and stained for 30 min on ice with the following antibodies (1 µl each per 10^6^ cells): rat anti-F4/80-FITC (clone BM8, eBioscience), rat anti-CD45-V500 (clone 30-F11, BD Biosciences), rat anti-CD11b-V450 (clone M1/70, BD Biosciences), rat anti-Ly6G-PerCP-Cy5.5 (clone 1A8, BD Biosciences), rat anti-CD3-PE (clone 17A2, BD Biosciences), rat-anti-CD19-PE-Cy7 (clone 1D3, BD Biosciences). Cells were analyzed with a BD FACSCanto II flow cytometer and FlowJo software (Tree Star).

### *In situ* hybridization

Tissue sections were analyzed by *in situ* hybridization as described previously [96]. Combined *in situ* hybridization and immunohistochemistry was performed according to a modified protocol [97].

### Immunostaining

Immunostaining was performed as described previously [53] using the following primary antibodies: rat anti-CD3 (clone 17A2, BD Biosciences, 1∶500), rat anti-Ly6G (clone 1A8, BD Biosciences, 1∶5000) and rabbit anti-S100A4 (Abcam, 1∶200).

### Stereology

Cells were counted using the optical fractionator method as described previously [53]. The counting parameters were as follows: distance between counting frames, 400×400 µm; counting frame size, 120×120 µm; dissector height, 10 µm; guard zone thickness, ≥2 µm.

### Microscopy

Micrographs were taken using a Retiga EX monochrome camera mounted on a Nikon E800 microscope. Images were adjusted for contrast, brightness and sharpness using Photoshop 12 (Adobe Systems).

### Statistics

Data are expressed as mean ± standard error and were analyzed using non-parametric tests, because they were discontinuous, were not normally distributed or the variances were not equal. Means were compared using the Wilcoxon or Kruskal-Wallis test. Correlation between qRT-PCR results was evaluated using the Spearman's test. EAE incidence curves were constructed using the Kaplan-Meier method and compared using the Wilcoxon test. All these analyses were performed using JMP 10 (SAS Institute) with a significance level of 5%.

## Supporting Information

Figure S1 Depletion of CD11b^+^ peritoneal leukocytes in CD11b-TK^mt-30^ mice by GCV treatment. Flow cytometric analysis of peritoneal leukocytes from CD11b-TK^mt-30^ mice treated twice daily for 6 days with GCV (50 mg/kg) or saline. All the animals were killed 6 h after injection of PTX (20 µg/kg). Cells were gated on CD45. *Significantly different from the saline group according to the Wilcoxon test (*P*≤0.0040). Sample size: 7 per group. Δ =  Difference compared to the saline group.
(TIF)

Figure S2 PTX increases the presence of DsRed-expressing myeloid cells in the peritoneum of pIL1-DsRed transgenic mice. ***a***, Flow cytometric analysis of peritoneal leukocytes harvested from pIL1-DsRed transgenic mice 6 h after intraperitoneal injection of PTX (20 µg/kg) or PBS. Cells were gated on CD45. ***b***, Representative examples of DsRed expression in peritoneal macrophages (CD11b^+^F4/80^+^), neutrophils (CD11b^+^Ly6G^+^), and non-myeloid leukocytes (CD11b^−^) exposed or not to PTX. Cells were gated as illustrated in ***a***. ***c***, Percentages of peritoneal leukocytes expressing DsRed in response to PTX. *Significantly different from the corresponding PBS group according to the Wilcoxon test (*P*≤0.008). Sample size: 5–6 per group.
(TIF)

Figure S3 NLRP3 does not mediate the effect of PTX on IL-1β and IL-6 expression. ***a–c***, Quantification of IL-6 and IL-1β by ELISA in plasma or peritoneal fluid from NLRP3-knockout mice killed 6 h after intraperitoneal injection of PTX (20 µg/kg) or PBS. *Significantly different from the PBS group according to Wilcoxon tests (*P*≤0.0034). Sample size: 5 (PBS) or 8 (PTX). ***d***, Quantification of IL-1β mRNA by qRT-PCR in peritoneal leukocytes from NLRP3-knockout mice treated or not with PTX. *Significantly different from the PBS group according to the Wilcoxon test (*P* = 0.0073). Sample size: 5 (PBS) or 7 (PTX).
(TIF)

Figure S4 Contrary to PTX, LPS stimulates IL-1β and IL-6 secretion in pyrin-deficient mice. Quantification of IL-1β and IL-6 by ELISA in peritoneal fluid collected from pyrin-deficient and wild-type mice 6 h after intraperitoneal injection of LPS (1 mg/kg), PTX (20 µg/kg) or PBS. *Significantly different from the corresponding wild types according to Wilcoxon tests (*P*<0.05). Sample size: 4–7 per group.
(TIF)

Figure S5 Neutrophil infiltration into the peritoneum after PTX exposure is not affected by the absence of pyrin. Percentage of peritoneal leukocyte subpopulations (i.e., Ly6G^+^CD11b^+^ neutrophils, F4/80^+^CD11b^+^ macrophages, CD19^+^CD11b^−^ B lymphocytes and CD3^+^CD11b^−^ T lymphocytes) in pyrin-deficient and wild-type mice 6 h after intraperitoneal injection of PTX (20 µg/kg) or PBS, as estimated by flow cytometry. Cells were gated on CD45. *Significantly different according to two-way ANOVA (*P*-values: overall, <0.0001; genotype effect, 0.49, treatment effect, <0.0001; interaction, 0.45. Sample size: 4–5 per group.
(TIF)

Figure S6 PTX induces the synthesis of IL-1β and pyrin in peritoneal leukocytes *ex vivo*, but not IL-1β processing and secretion through the pyrin inflammasome. ***a,b***, Quantification of IL-1β by ELISA in cell extracts and supernatants from peritoneal leukocytes, which were isolated from wild-type or pyrin-knockout mice and incubated for 6 h with PTX (20 µg/kg), heat-inactivated PTX (PTX^hi^), mutant PTX (PTX^mut^) or PBS. *Significantly different from the corresponding PBS group according to *post hoc* Wilcoxon tests (Kruskal-Wallis test, *P*≤0.02). Sample size: 3–5 per group. ***c***, Bivariate analysis showing a positive correlation between the amounts of pyrin and IL-1β mRNAs (estimated by qRT-PCR) in peritoneal leukocytes stimulated *ex vivo* (Spearman's test, *P*<0.0001, *R* = 0.94).
(TIF)

Table S1 Mice used in this study.
(PDF)
