# Supplementary figures and images for: The Inflammasome Pyrin Contributes to Pertussis Toxin-Induced IL-1β Synthesis, Neutrophil Intravascular Crawling and Autoimmune Encephalomyelitis
Source: PLoS Pathog. 2014 May 29;10(5):e1004150. doi: 10.1371/journal.ppat.1004150 (PMC4038594; doi:10.1371/journal.ppat.1004150)

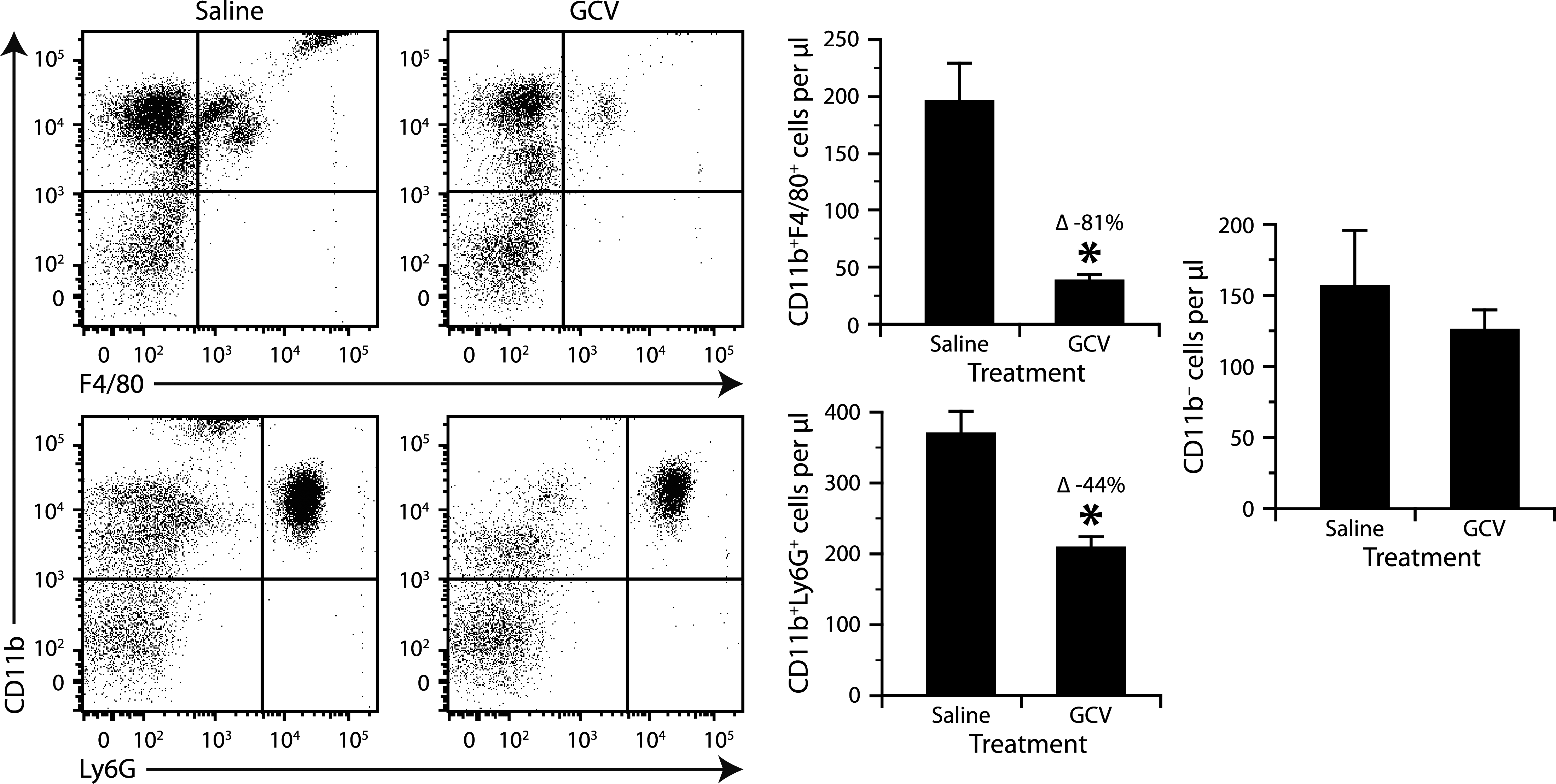

Supplement: Figure S1 — Depletion of CD11b+ peritoneal leukocytes in CD11b-TKmt-30 mice by GCV treatment. Flow cytometric analysis of peritoneal leukocytes from CD11b-TKmt-30 mice treated twice daily for 6 days with GCV (50 mg/kg) or saline. All the animals were killed 6 h after injection of PTX (20 µg/kg). Cells were gated on CD45. *Significantly different from the saline group according to the Wilcoxon test (P≤0.0040). Sample size: 7 per group. Δ = Difference compared to the saline group. (TIF) [file ppat.1004150.s001.tif]

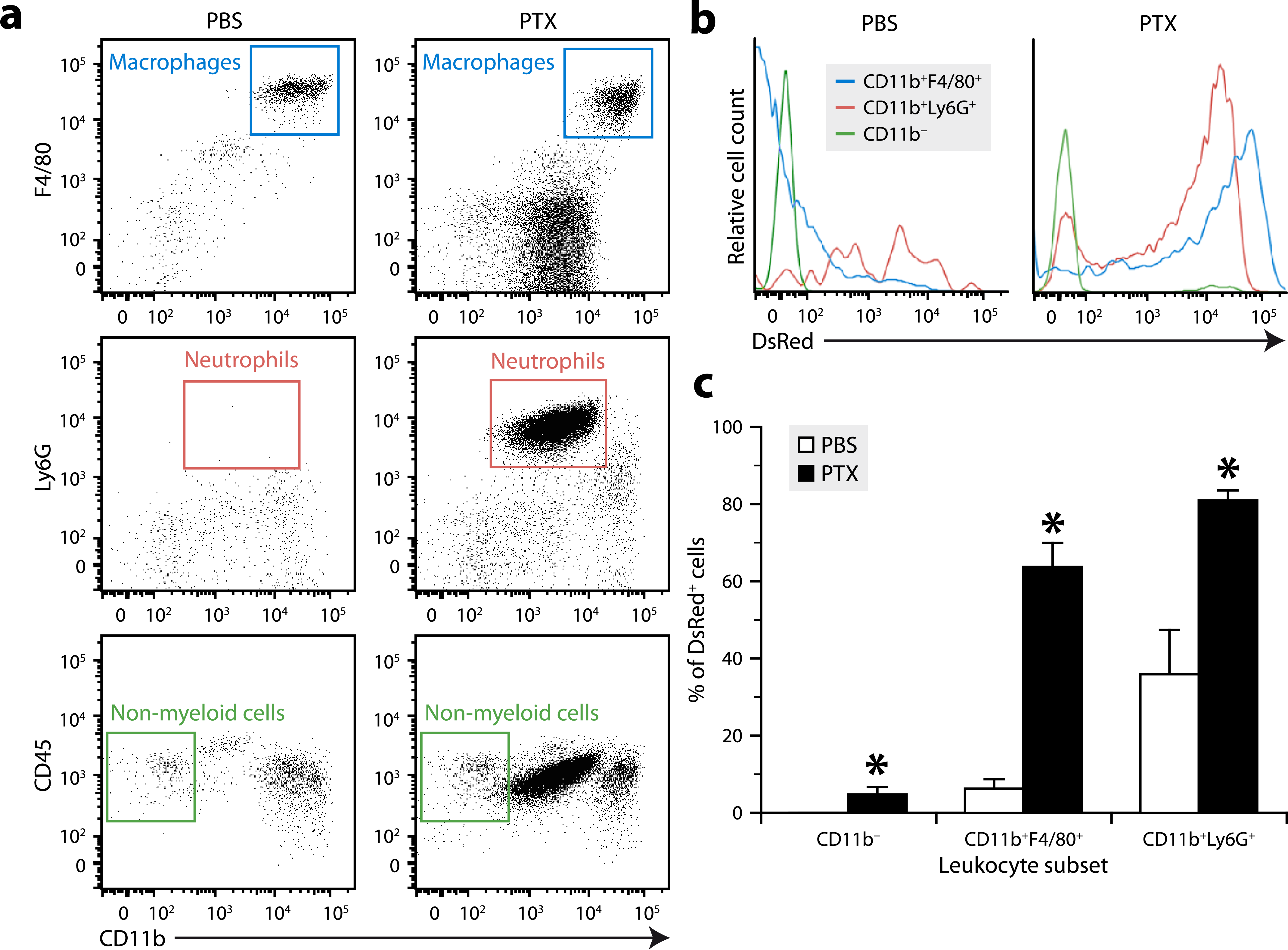

Supplement: Figure S2 — PTX increases the presence of DsRed-expressing myeloid cells in the peritoneum of pIL1-DsRed transgenic mice. a, Flow cytometric analysis of peritoneal leukocytes harvested from pIL1-DsRed transgenic mice 6 h after intraperitoneal injection of PTX (20 µg/kg) or PBS. Cells were gated on CD45. b, Representative examples of DsRed expression in peritoneal macrophages (CD11b+F4/80+), neutrophils (CD11b+Ly6G+), and non-myeloid leukocytes (CD11b−) exposed or not to PTX. Cells were gated as illustrated in a. c, Percentages of peritoneal leukocytes expressing DsRed in response to PTX. *Significantly different from the corresponding PBS group according to the Wilcoxon test (P≤0.008). Sample size: 5–6 per group. (TIF) [file ppat.1004150.s002.tif]

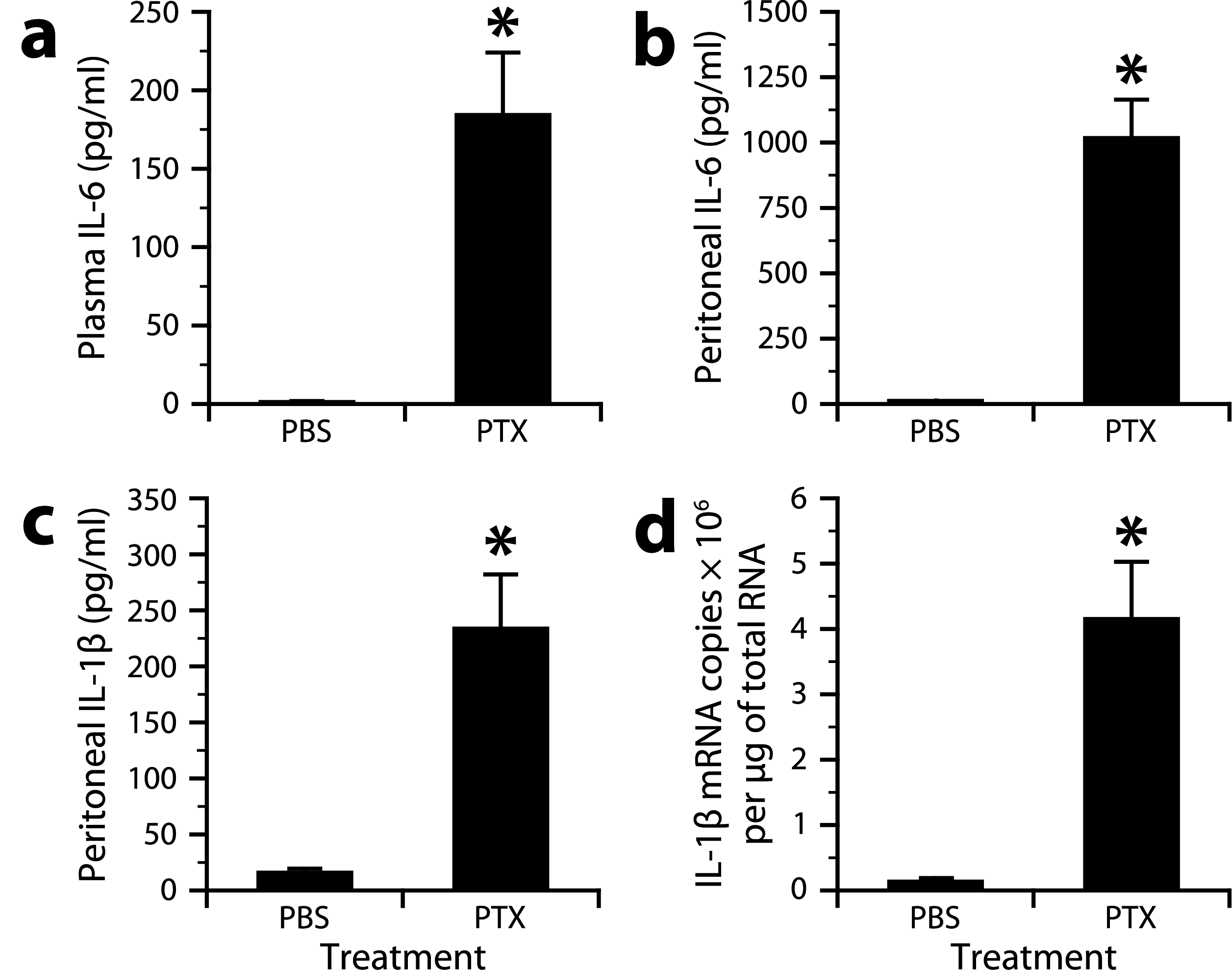

Supplement: Figure S3 — NLRP3 does not mediate the effect of PTX on IL-1β and IL-6 expression. a–c, Quantification of IL-6 and IL-1β by ELISA in plasma or peritoneal fluid from NLRP3-knockout mice killed 6 h after intraperitoneal injection of PTX (20 µg/kg) or PBS. *Significantly different from the PBS group according to Wilcoxon tests (P≤0.0034). Sample size: 5 (PBS) or 8 (PTX). d, Quantification of IL-1β mRNA by qRT-PCR in peritoneal leukocytes from NLRP3-knockout mice treated or not with PTX. *Significantly different from the PBS group according to the Wilcoxon test (P = 0.0073). Sample size: 5 (PBS) or 7 (PTX). (TIF) [file ppat.1004150.s003.tif]

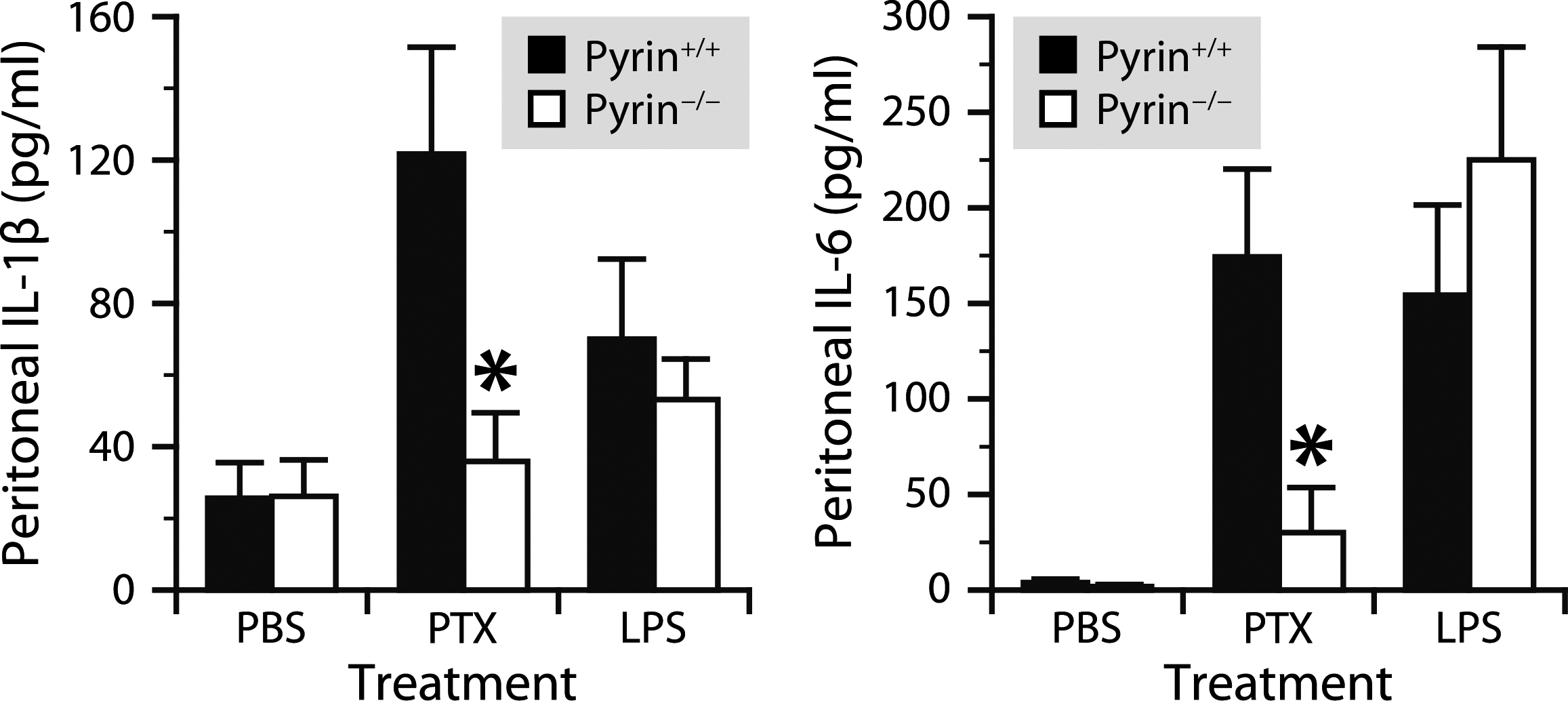

Supplement: Figure S4 — Contrary to PTX, LPS stimulates IL-1β and IL-6 secretion in pyrin-deficient mice. Quantification of IL-1β and IL-6 by ELISA in peritoneal fluid collected from pyrin-deficient and wild-type mice 6 h after intraperitoneal injection of LPS (1 mg/kg), PTX (20 µg/kg) or PBS. *Significantly different from the corresponding wild types according to Wilcoxon tests (P<0.05). Sample size: 4–7 per group. (TIF) [file ppat.1004150.s004.tif]

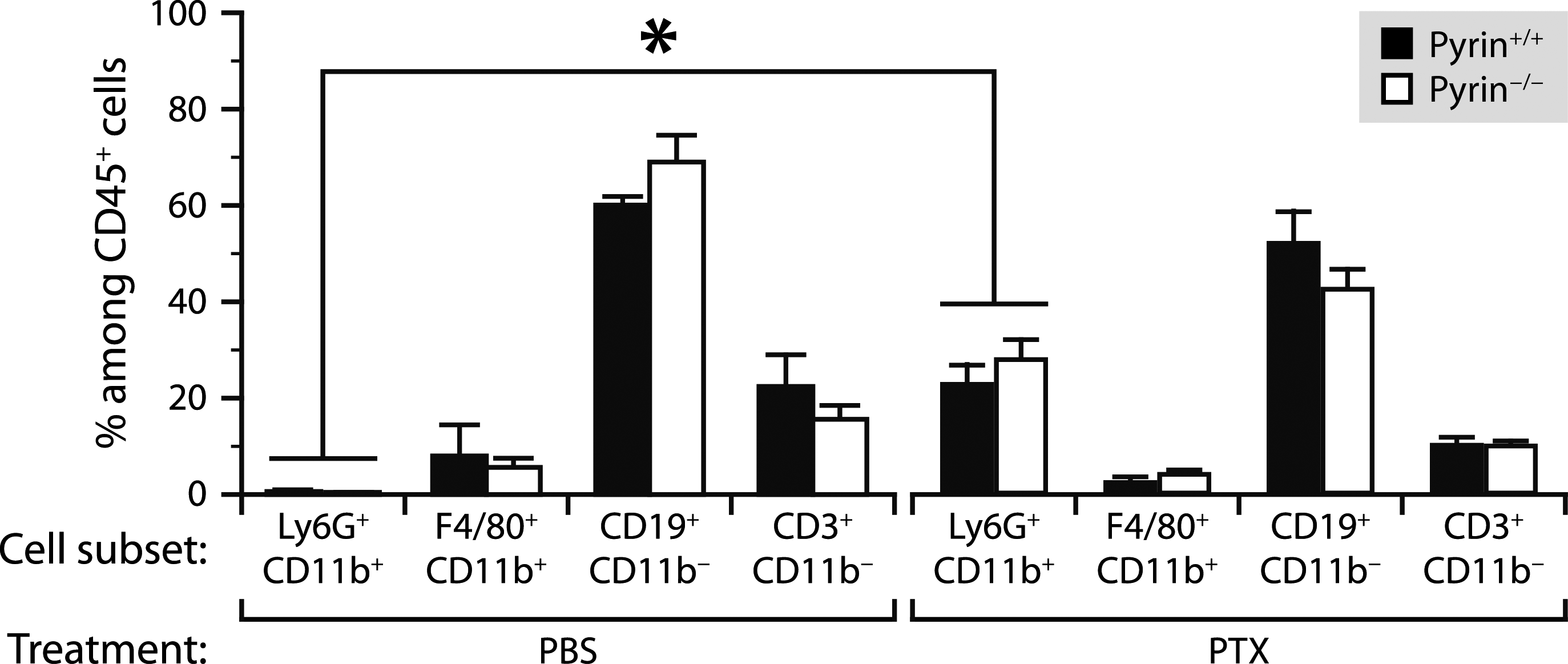

Supplement: Figure S5 — Neutrophil infiltration into the peritoneum after PTX exposure is not affected by the absence of pyrin. Percentage of peritoneal leukocyte subpopulations (i.e., Ly6G+CD11b+ neutrophils, F4/80+CD11b+ macrophages, CD19+CD11b− B lymphocytes and CD3+CD11b− T lymphocytes) in pyrin-deficient and wild-type mice 6 h after intraperitoneal injection of PTX (20 µg/kg) or PBS, as estimated by flow cytometry. Cells were gated on CD45. *Significantly different according to two-way ANOVA (P-values: overall, <0.0001; genotype effect, 0.49, treatment effect, <0.0001; interaction, 0.45. Sample size: 4–5 per group. (TIF) [file ppat.1004150.s005.tif]

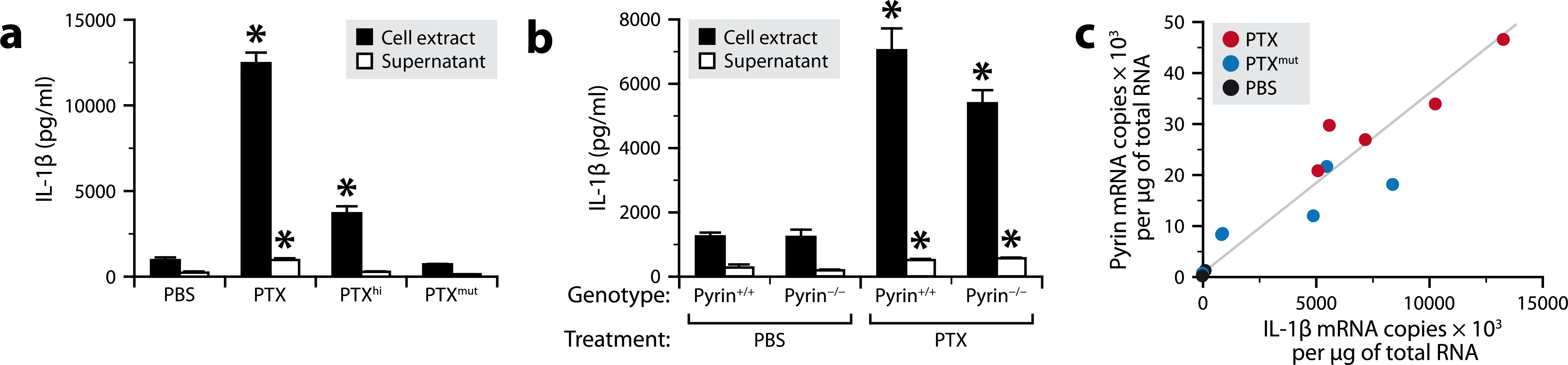

Supplement: Figure S6 — PTX induces the synthesis of IL-1β and pyrin in peritoneal leukocytes ex vivo, but not IL-1β processing and secretion through the pyrin inflammasome. a,b, Quantification of IL-1β by ELISA in cell extracts and supernatants from peritoneal leukocytes, which were isolated from wild-type or pyrin-knockout mice and incubated for 6 h with PTX (20 µg/kg), heat-inactivated PTX (PTXhi), mutant PTX (PTXmut) or PBS. *Significantly different from the corresponding PBS group according to post hoc Wilcoxon tests (Kruskal-Wallis test, P≤0.02). Sample size: 3–5 per group. c, Bivariate analysis showing a positive correlation between the amounts of pyrin and IL-1β mRNAs (estimated by qRT-PCR) in peritoneal leukocytes stimulated ex vivo (Spearman's test, P<0.0001, R = 0.94). (TIF) [file ppat.1004150.s006.tif]
