# Supplementary material for: The Inflammasome Pyrin Contributes to Pertussis Toxin-Induced IL-1β Synthesis, Neutrophil Intravascular Crawling and Autoimmune Encephalomyelitis
Source: PLoS Pathog. 2014 May 29;10(5):e1004150. doi: 10.1371/journal.ppat.1004150 (PMC4038594; doi:10.1371/journal.ppat.1004150)
Supplement: Table S1 — Mice used in this study. (PDF) [file ppat.1004150.s007.pdf]

Table S1. Mice used in this study.

Name	Source	Genetic background	Reference (Pubmed ID)
2D2	The Jackson Laboratory (Cat#006912)	C57BL/6	12732654
ASC$^{-/-}$	Dr. Vishva Dixit, Genentech	C57BL/6	15190255
C3HeB/Fe	The Jackson Laboratory (Cat#000658)	C3H	-
C57BL/6	The Jackson Laboratory (Cat#000664)	C57BL/6	-
CD11b-TK$^{mt\text{-}30}$	Dr. Jean-Pierre Julien	C57BL/6	16276506
Casp1$^{-/-}$	The Jackson Laboratory (Cat#004947)	NOD/ShiLt	7535475
IL-1β$^{-/-}$	Dr. Yoichiro Iwakura, University of Tokyo	C57BL/6	9565638
IL-6$^{-/-}$	The Jackson Laboratory (Cat#002650)	C57BL/6	8127368
NLRP3$^{-/-}$	Dr. Vishva Dixit, Genentech	C57BL/6	16407890
NLRP6$^{-/-}$	Dr. Grace Chen, University of Michigan	C57BL/6	21543645
NOD/ShiLt	The Jackson Laboratory (Cat#001976)	NOD/ShiLt	-
pIL1-DsRed	Dr. Akira Takashima	C57BL/6	20147964
Pyrin$^{-/-}$	Dr. Daniel Kastner, NHGRI	C57BL/6	21600797
TLR4$^{-/-}$ (C3H/He)	The Jackson Laboratory (Cat#000659)	C3H	9851930
